# Bone-Marrow-Derived Mesenchymal Stromal Cells (MSC) from Diabetic and Nondiabetic Rats Have Similar Therapeutic Potentials

**DOI:** 10.5935/abc.20170176

**Published:** 2017-12

**Authors:** Vitória Santório de São José, Gustavo Monnerat, Barbara Guerra, Bruno Dias Paredes, Tais Hanae Kasai-Brunswick, Antonio Carlos Campos de Carvalho, Emiliano Medei

**Affiliations:** Universidade Federal do Rio de Janeiro (UFRJ), Rio de Janeiro, RJ - Brazil

**Keywords:** Diabetes Mellitus, Mesenchymal Stromal Cells, Cardiac Electrophysiology, Cell and Tissue-Based Therapy, Rats

## Abstract

**Background:**

Diabetes mellitus is a severe chronic disease leading to systemic complications, including cardiovascular dysfunction. Previous cell therapy studies have obtained promising results with the use bone marrow mesenchymal stromal cells derived from healthy animals (MSCc) in diabetes animal models. However, the ability of MSC derived from diabetic rats to improve functional cardiac parameters is still unknown.

**Objectives:**

To investigate whether bone-marrow-derived MSC from diabetic rats (MSCd) would contribute to recover metabolic and cardiac electrical properties in other diabetic rats.

**Methods:**

Diabetes was induced in Wistar rats with streptozotocin. MSCs were characterized by flow cytometry, morphological analysis, and immunohistochemistry. Cardiac electrical function was analyzed using recordings of ventricular action potential. Differences between variables were considered significant when p < 0.05.

**Results:**

In vitro properties of MSCc and MSCd were evaluated. Both cell types presented similar morphology, growth kinetics, and mesenchymal profile, and could differentiate into adipogenic and osteogenic lineages. However, in an assay for fibroblast colony-forming units (CFU-F), MSCd formed more colonies than MSCc when cultured in expansion medium with or without hydrocortisone (1 µM). In order to compare the therapeutic potential of the cells, the animals were divided into four experimental groups: nondiabetic (CTRL), diabetic (DM), diabetic treated with MSCc (DM + MSCc), and diabetic treated with MSCd (DM + MSCd). The treated groups received a single injection of MSC 4 weeks after the development of diabetes. MSCc and MSCd controlled hyperglycemia and body weight loss and improved cardiac electrical remodeling in diabetic rats.

**Conclusions:**

MSCd and MSCc have similar in vitro properties and therapeutic potential in a rat model of diabetes induced with streptozotocin.

## Introduction

Diabetes mellitus is a metabolic disease mainly characterized by chronic hyperglycemia leading to several complications in different organs and systems.^1^ Despite insulin therapy, exercise programs, and nutritional interventions, most patients with diabetes are unable to maintain blood glucose levels within the normal range, resulting in pathological complications. Even though diabetes is a systemic disease, cardiac complications are the main cause of morbidity and mortality related to this condition. Over 75% of all deaths in patients with diabetes are caused by cardiovascular complications.^2-4^ In this context, diabetic cardiomyopathy leads to cardiac electrical abnormalities, including increased QT and QTc intervals and QT dispersion. Indeed, these proarrhythmic electrocardiographic abnormalities are triggered by a prolongation of the action potential (AP) duration as a consequence of the cardiac electrical remodeling that occurs in the disease.^5-9^

A small population of mononuclear bone marrow cells named mesenchymal stromal cells (MSC) is an attractive source of cells to treat diabetes and related cardiovascular complications.^7,10,11^ MSC have clonogenic potential^12^ and under specific culture conditions are able to differentiate into cells of various mesenchymal lineages such as osteoblasts, chondrocytes, and adipocytes.^13,14^ Additionally, MSC are multipotent stem cells with immunomodulatory properties able to regulate several physiological responses.^15^ Inflammation control is a potential therapeutic intervention for several autoimmune diseases, as well as sterile inflammatory processes such as diabetes.^15-20^

The mechanisms underlying the therapeutic effects of MSC are mainly due to their secretion of paracrine factors, stimulated by hyperglycemic and inflammatory microenvironments. Indeed, MSC have been described as secreting cardioprotective factors that may improve cardiac function.^15,16,21-24^ Additionally, the therapeutic potential of MSC in rescuing metabolic control in mice with diabetes has been demonstrated.^25,26^ A previous study from our group has shown that rats with diabetes transplanted with bone marrow MSC derived from healthy rats (MSCc) showed improved metabolic regulation and reversal of diabetes-induced cardiac electrical and mechanical abnormalities through systemic immunomodulation.^7^

Several studies have reported that bone marrow MSC derived from healthy donors are beneficial in the treatment of diabetes, based on the immunomodulatory and regenerative properties of these cells. However, the possibility of MSC autotransplantation would simplify the clinical application of this cell therapy in patients with diabetes, considering that autologous cell transplantation minimizes complications for the recipient such as rejection, immunosuppressive treatment, and transmission of infectious agents.

Based on this clinical perspective, the purpose of the present work was to verify whether the transplantation of bone marrow MSC from donor diabetic rats (MSCd) could contribute to recovering the metabolic and cardiac functions of other diabetic rats. In order to achieve that, a comparative study between MSCc and MSCd derived from rats was performed.

## Methods

### Animals and protocols

**Figure 1:**
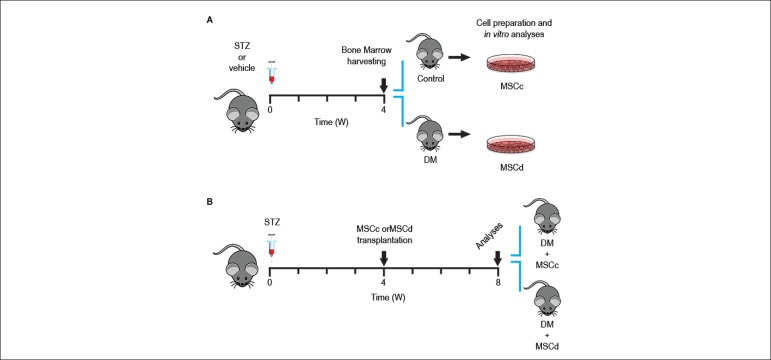
Experimental study design. (A) Animals were treated with vehicle and streptozotocin (STZ) at week 0. Bone marrow cells were harvested on the 4th week for culture and in vitro analyses. (B) MSC therapy scheme. Diabetic animals (STZ) were divided into two groups: the first one received 5 x 106 cells from healthy rats (DM + MSCc) and the second received the same quantity of cells from diabetic rats (DM + MSCd) after 4 weeks of disease. The cells were injected into the animals’ retroocular plexus. Blood glucose levels and body weight were evaluated during 4 weeks after transplantation. At the end of the protocol, the animals were sacrificed, and their hearts were isolated for potential recording.

All animal procedures were carried out in accordance with the Guide for the Care and Use of Laboratory Animals (National Institutes of Health [NIH], USA) and approved by our local institutional committee under the number IBCCF 217-09/16. In this study, 1-month-old male Wistar rats (100 ± 20 g) were housed under controlled temperature (23 ± 2ºC) with 12-hour light cycles and fed standard chow and water *ad libitum*. Diabetes was induced as described below. Four weeks after diabetes was established, the animals were divided into four experimental groups: (a) nondiabetic rats receiving only the vehicle (CTRL; n = 7); (b) diabetic rats receiving only the vehicle (DM; n = 7), (c) diabetic rats transplanted with bone marrow MSC derived from healthy rats (DM + MSCc; n = 7); and (4) diabetic rats transplanted with bone marrow MSC derived from other diabetic rats (DM + MSCd; n = 8). Both vehicle and cells were injected into the animals’ retroocular plexus. Body weight and blood glucose levels were measured weekly during 4 weeks after cell therapy in all four experimental groups. Figure 1 illustrates the study design.

### Diabetes induction and glucose measurement

Diabetes was induced with a single intravenous streptozotocin dose (Sigma-Aldrich, USA) 80 mg/kg diluted in citrate buffer (0.05 M) in anesthetized animals (isoflurane, Baker Norton, UK).^7^ Nondiabetic animals (CTRL group) received only the vehicle. At 72 hours after induction, the animals fasted for 5 hours during early morning and had their blood glucose levels measured. Only animals with glucose levels higher than 250 mg/dL were considered to have diabetes. Blood glucose levels were analyzed with a standard glucometer (Contour TS, Bayer HealthCare LLC, USA). The blood samples were obtained from the tail vein of unanesthetized animals.

### Mesenchymal stromal cell: isolation and culture

The animals were anesthetized and then sacrificed by cervical dislocation. Their legs were cleaned with alcohol 70%, and their tibial and femoral bones were isolated and centrifuged at 1,000 xg for 3 min at 4ºC for collection of marrow cells. The cell suspension was diluted in phosphate buffered saline (PBS) and centrifuged at 300 xg for 5 min at 4ºC. Pelleted cells were diluted in Dulbecco’s modified Eagle medium (DMEM, Gibco-Invitrogen, Carlsbad, CA, USA) and centrifuged in Histopaque gradient (1.083 g/mL, Sigma-Aldrich, USA) at 400 xg for 30 min. Mononuclear cells were collected from the interface and washed three times with PBS, and their viability was checked using 0.4 % trypan blue solution (Sigma-Aldrich). Finally, the cells were plated at a density of 1.2 x 10^6^cells/cm^2^ and maintained at 37ºC in a 5% CO_2_ atmosphere. The medium (DMEM supplemented with 20% fetal bovine serum [Gibco-Invitrogen, USA] and penicillin G/streptomycin 1% [Gibco-Invitrogen]) was changed twice weekly, preserving only the adherent cells. When 80-90% confluence was reached, the adherent cells were detached from the culture plates with 0.25% trypsin-EDTA (Sigma-Aldrich) and expanded at a density of 1.2 x 10^4^ cells/cm^2^ until passage 3, as previously described.^7^

### Immunophenotypic characterization

For the immunophenotypic analysis, third passage MSCc and MSCd were dissociated and suspended in blocking solution containing cold PBS supplemented with 0.5% bovine serum albumin (BSA). The cells were treated with rat Fc block (CD32, Cat#550271; BD Biosciences, San Jose, CA, USA) for 20 minutes before incubation with antibodies. The following antibodies conjugated with fluorescein isothiocyanate (FITC), phycoerythrin (PE), or biotin were used: CD29 (clone Ha2/5, Cat#555005, dilution 1:50; BD Biosciences), CD90-1 (clone OX-7, Cat#551401, dilution 1:25; BD Biosciences), CD45 (clone 30-F11, Cat#553077, dilution 1:50; BD Biosciences), and CD34 (clone RAM34, Cat#551387, dilution 1:50; BD Biosciences). The corresponding isotypes (BD Biosciences) were used as nonspecific binding controls. After incubation at 4ºC for 20 minutes, the cells were washed with PBS/0.5% BSA, centrifuged at 300 xg for 5 minutes, and suspended in PBS for data acquisition. DAPI 0.1 µg/mL (Cat# D9452, Sigma-Aldrich) was added to exclude dead cells. The samples were acquired in a BD FACSAria II flow cytometer (BD Biosciences) and the resulting data were analyzed using the software FlowJo, version 10.1.

### Adipogenic and osteogenic differentiation

Third passage MSCc (n = 3) and MSCd (n = 3) were plated in a 6-well plate at 3 x 10^4^ cells per well. The media was changed twice a week, and no passages were made during the differentiation protocols. In order to induce adipogenic differentiation, the expansion medium was supplemented with dexamethasone 1 µM (Sigma-Aldrich), IBMX 0.5 mM (Sigma-Aldrich), insulin 10 µg/mL (Sigma-Aldrich), and indomethacin 200 µM (Vetec Química Fina, Duque de Caxias, RJ, Brazil). The cells were incubated at 37 ºC in a 5% CO_2_ atmosphere for 21 days and then fixed with 4% paraformaldehyde for 15 minutes, while cytoplasmic lipid droplets were stained with 0.5% oil red O. Osteogenic differentiation was performed by culturing MSCc and MSCd in expansion medium supplemented with dexamethasone 1 µM (Sigma-Aldrich), β-glycerophosphate 10 mM (Sigma-Aldrich), and ascorbic acid 0.5 µM (Sigma-Aldrich) for 21 days. The cells were then fixed with 4% paraformaldehyde for 15 minutes, and extracellular calcium deposits were stained after incubation with 1% alizarin red in water.

### Population doubling time (PDT)

For evaluation of growth kinetics, MSCc and MSCd (passage 3) were cultured in 35-mm cell culture dishes with a 2-mm grid (Sarstedt, Newton, NC, USA) at a density of 1.2 x 10^3^ cells/cm^2^. The cells were maintained in expansion medium and incubated at 37ºC in a 5% CO_2_ atmosphere for 7 days. Four random grids were counted daily, and the mean number of cells per mm^2^ was calculated. The obtained values were used to build a cell/mm^2^ *versus* time curve. Linear regression was performed with base-2 logarithm transformation of the cell/mm^2^ axis, in which the inverse of the angular coefficient α was used to calculate the PDT.

### Fibroblast colony-forming units (CFU-F)

In order to perform this experiment, freshly isolated mononuclear cells derived from diabetic (MSCd; n = 3) and nondiabetic (MSCc; n = 3) rats were isolated and seeded into 6-well plates at a density of 2.08 x 10^5^ cells/cm^2^ in each well. The cells were cultured in expansion medium with and without hydrocortisone 1 µM. After 16 days, the cells were fixed with methanol PA (Vetec Química Fina) for 5 minutes, and the number of colonies was counted manually after Giemsa (Merck, Darmstadt, Germany) staining.

### Cell therapy protocol

Diabetic rats were transplanted with 5 x 10^6^ MSCc from healthy rats (DM + MSCc; n = 7) or 5 x 10^6^ MSCd from diabetic rats (DM + MSCd; n = 8). The cells were transplanted into the retroocular plexus (200 L). Blood glucose levels and body weight were evaluated for 4 weeks after the transplantation. At the end of the protocol, the animals were sacrificed, and their hearts were isolated for AP recording. Control and diabetic rats received retroocular injections with the same volume of saline solution.

### Action potential recording

For AP recording, left ventricular cardiac muscle strips were obtained and pinned to the bottom of a Sylgard-coated tissue bath to expose the endocardial side. The strips were continuously perfused with oxygenated Tyrode solution at 37ºC. The composition of the Tyrode solution (mM) was: 150.8 NaCl, 5.4 KCl, 1.8 CaCl_2_, 1.0 MgCl_2_, 11.0 D-glucose, and 10.0 HEPES (pH 7.4 adjusted with NaOH at 37.0 ± 0.5ºC). The tissue was stimulated at a basic cycle length of 1,000 ms. The transmembrane potential was recorded using glass microelectrodes (10-40 MΩ DC resistance) filled with 2.7 M KCl, connected to a high input impedance microelectrode amplifier (MEZ7200, Nihon Kohden, Japan). Amplified signals were digitized (1440 Digidata A/D interface, Axon Instrument, Inc., Sunnyvale, USA) and stored in a computer for later analysis using the software LabChart, 7.3 (ADInstruments, Bella Vista, Australia). The following AP parameters were analyzed: resting membrane potential, AP amplitude (APA), and AP duration at 90% of repolarization (APD_90_), as previously described.^27^

### Statistical analysis

Values are expressed as mean ± standard deviation (SD). For *in vitro* assays, comparisons between MSCc and MSCd were performed using unpaired Student’s *t* test, and for *in vivo* analysis, analysis of variance (ANOVA) was used, followed by the Bonferroni test for multiple comparisons. Data showing non-Gaussian distribution (Kolmogorov-Smirnov test) were compared by the Kruskal-Wallis test followed by Dunn’s multiple comparison test. Differences between variables were considered significant when p < 0.05. All analyses were performed using GraphPad Prism 5.0 (GraphPad Software, San Diego, CA, USA). The sample size was not predetermined with statistical methods and was estimated on the basis of sample availability and previous experimental cardiovascular studies using stem cell treatment.^7^

## Results

### MSCc and MSCd morphology and surface phenotype

**Figure 2:**
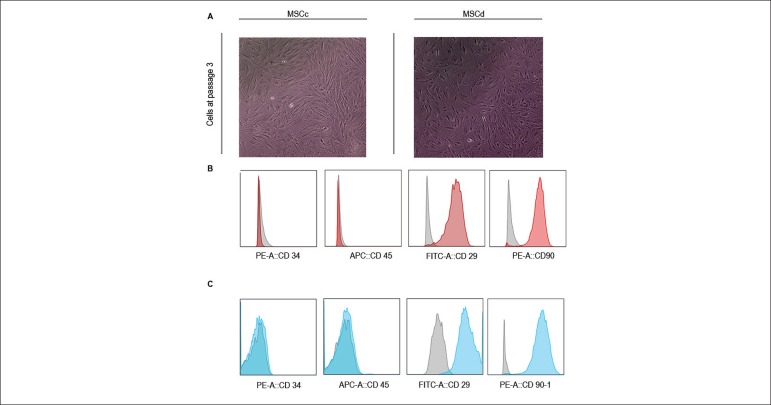
Characterization of MSC profile on third passage. (A) Similar fibroblast-like morphology of MSCc and MSCd 4 days after the cells were seeded onto culture dishes. (B) Flow cytometry analysis of MSC derived from healthy animals (MSCc) and (C) MSC derived from diabetic animals (MSCd). Color histograms showing high expression of mesenchymal surface proteins (CD29 and CD90) and low expression of hematopoietic markers (CD34 and CD45). The gray histograms represent the isotype controls.

MSCc and MSCd adhered to plastic and presented a fibroblast-like morphology 3-4 days after being seeded onto culture flasks. Nonadherent cells seen on primary cultures were discarded with media changes. Figure 2A shows third passage MSCc and MSCd. The mesenchymal profile of MSCc and MSCd was evaluated by surface expression of key markers on third passage cells by flow cytometry. Both types of cells were positive for the MSC-related markers (CD29 and CD90, > 90%) and negative for hematopoietic markers (CD45 and CD34, ≤ 2.5%) (Figures 2B and 2C). MSCc and MSCd phenotypes were similar.

### Adipogenic and osteogenic differentiation

**Figure 3:**
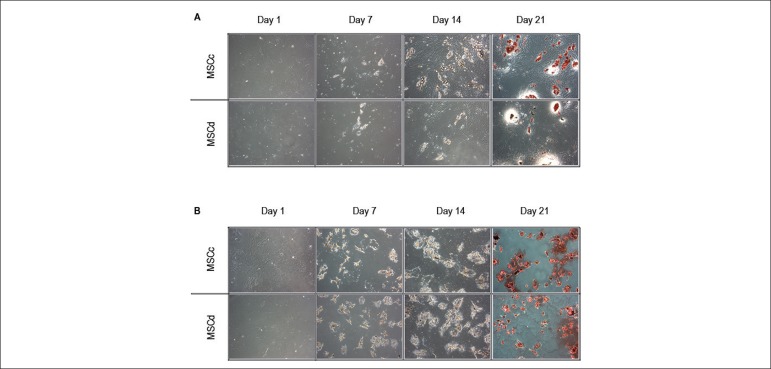
Adipogenic and osteogenic differentiation assays. The adipogenic and osteogenic differentiation assays were performed during 21 days using MSCc and MSCd. We started the protocol with 60% confluence culture. (A) In the adipogenic assay, small lipid droplets were present in both MSCc and MSCd at day 7 and increased in volume and number on day 14. At day 21, the cultures were stained with oil red O, and the lipid droplets were stained in red. Both MSCc and MSCd differentiated into adipocytes at the end of the protocol. (B) In the osteogenic assay, we observed dramatic morphologic changes starting at day 7 in both MSCc and MSCd, accentuating at day 14. At day 21, the cultures were stained with alizarin red and the calcium deposits stained in red. Both MSCc and MSCd generated a mineralized matrix at the end of the protocol.

During the adipogenic differentiation process, lipid droplet formation was already observed in the first week (Figure 3A). Adipogenic differentiation remained for 21 days. During this period, the droplets showed increasing volume and number. At the end of the protocol, lipid-rich vacuoles were stained with oil red O.

Osteogenic differentiation of both MSCc and MSCd also started during the first week (Figure 3B). Both experimental groups showed fast morphologic changes, which remained until 21 days later, at the end of the experiment. At 21 days after alizarin red staining, calcium deposits were observed, characterizing matrix mineralization.

As controls for both differentiation protocols, cultures of cells were obtained from diabetic and healthy animals and cultivated with expansion medium during the entire protocol and, as expected, showed no formation of lipid vacuoles or calcium deposits.

### Growth kinetics

**Figure 4:**
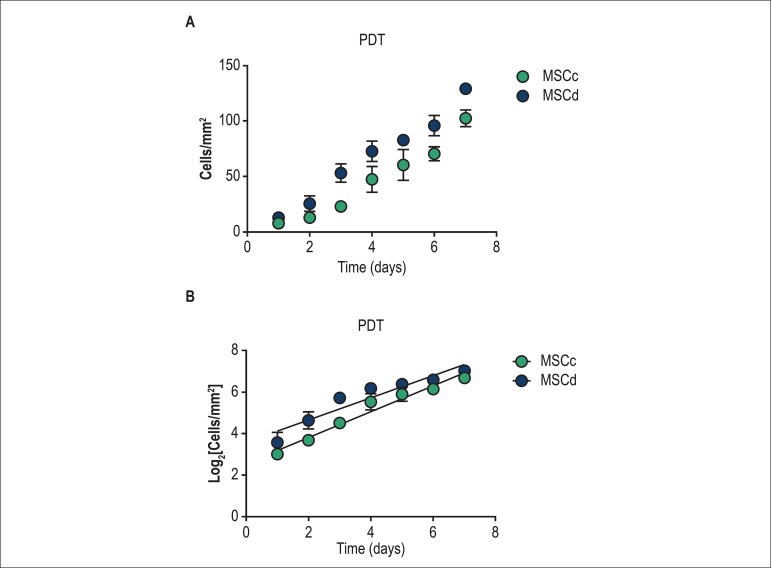
Growth kinetic evaluation by population doubling time (PDT) assay. (A) The number of MSC was counted daily (cells/mm^2^) after plating 104 cells at day zero for 7 days. (B) Linear regression of logarithmic cell growth (cells/mm^2^). There was no statistic difference between groups. The results are represented as mean ± standard deviation (SD). Abbreviations: MSCc - MSC derived from healthy animals; MSCd - MSC: derived from diabetic animals.

In order to evaluate the growth kinetics of MSCc and MSCd, the cells were plated in dishes and observed until reaching 100% confluence on day 7. The proliferation rate was determined by daily monitoring of the culture dishes (Figure 4A). As shown in Figure 4B, MSC from both healthy and diabetic rats showed similar grow kinetic characteristics.

### Clonogenic properties

**Figure 5:**
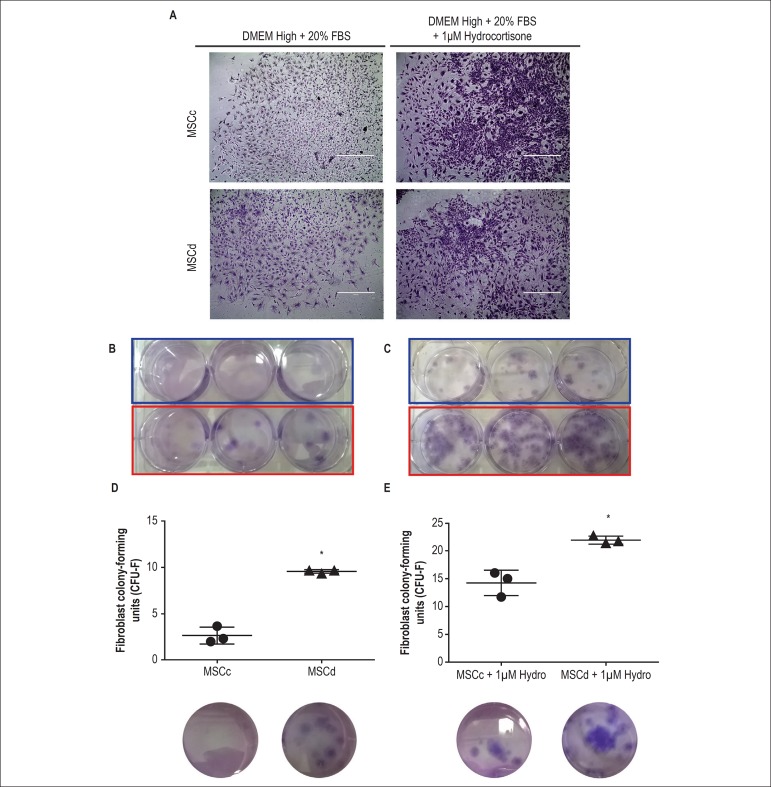
Fibroblast colony-forming units (CFU-F) assay. (A) Representative photomicrographs of colonies from MSCc and MSCd obtained after CFU-F assay using two different conditions, with and without hydrocortisone. (B) Macroscopic view of CFU-F formation after cells were cultured with no hydrocortisone condition. MSCc formed fewer CFU-F colonies than did MSCd. (C) Macroscopic view of CFU-F formation after cells were cultured with a 1 *µ*M hydrocortisone condition. Once again, MSCc formed fewer CFU-F colonies than did MSCd. (D-E) Quantitative comparisons between the numbers of CFU-F colonies forming a greater number in MSCd than MSCc in both conditions. The results are represented as mean ± standard deviation (SD) and * represents p < 0.05.

In order to assess the capacity of colony formation of each group, CFU-F assays of MSCc and MSCd in DMEM/high in the presence and absence of hydrocortisone (1 µM) were performed. Both cell types were able to generate CFU-F under both conditions (Figures 5A and 5B). However, the number of colonies was greater in the cultures with medium supplemented with hydrocortisone in both experimental groups (Figure 5B). Additionally, MSCd formed more CFU-F than MSCc, regardless of the protocol used (Figures 5D and 5E).

### Metabolic improvement after MSC transplantation

**Figure 6:**
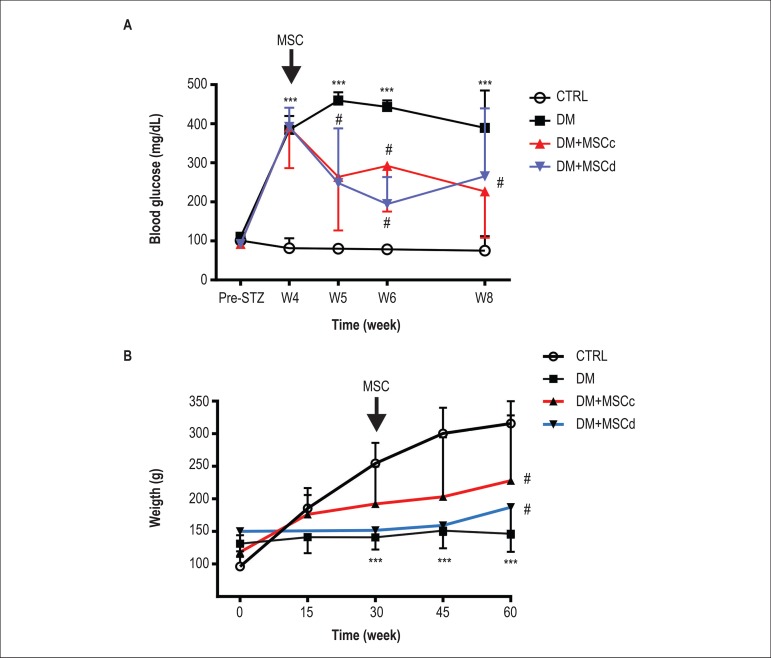
Metabolic profile after MSC transplantation. (A) Blood glucose levels were monitored during the 8 weeks of the protocol. Both cell types improved glucose levels compared with placebo but were unable to restore glucose levels to those of control animals. (B) Body weight improvement through the 60 days of the protocol. Both cell types were able to increase the animals’ body weight 30 days after cell injection when compared with placebo but were unable to restore their weights to those of control animals. Results are expressed as mean ± standard deviation (SD), *** represents p < 0.001 versus CTRL; # represents p < 0.05 versus DM and p < 0.01 versus CTRL.

In order to assess the therapeutic potential of both cell types, 4 weeks after establishing the diabetic rat model, MSCc or MSCd (5 x 10^6^ cells) were transplanted with a single injection into diabetic rats. MSCd and MSCc showed similar therapeutic potentials, with improvement of glucose levels (Figure 6A). In both cell-treated groups, blood glucose levels were lower than those in the placebo group but higher than those in rats without diabetes. Body weight loss in the diabetic groups was also partially rescued by both MSCc and MSCd transplantation (Figure 6B).

### MSCd improves the cardiac action potential profile

**Figure 7:**
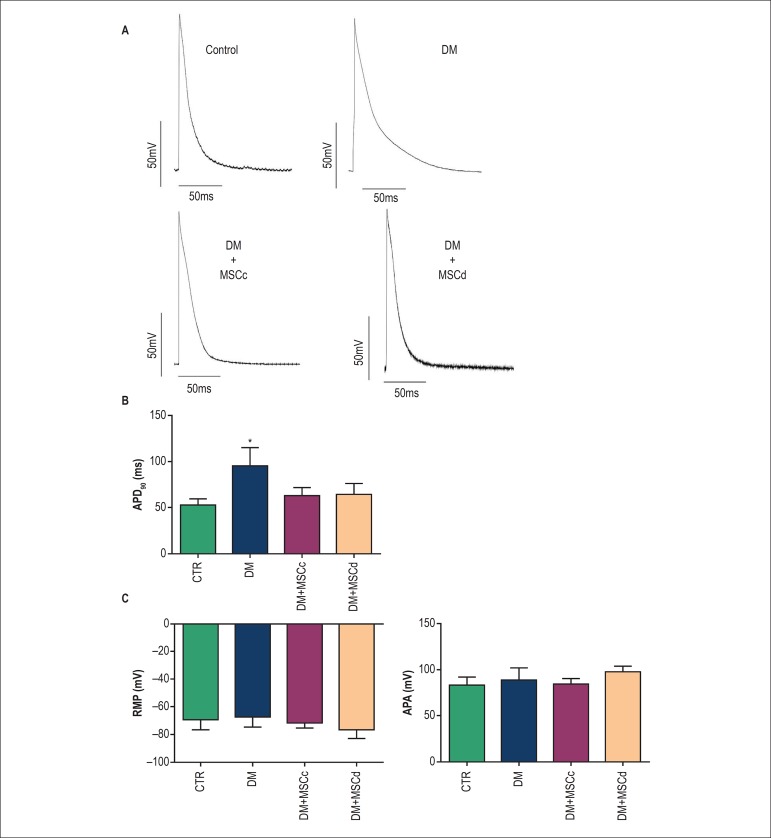
MSCc and MSCd improved cardiac remodeling 4 weeks after MSC transplantation. (A) Representative action potential traces from left ventricle endocardial tissue for all experimental groups. (B) Action potential duration at 90% repolarization (APA90). (C) Resting membrane potential (RMP) and action potential amplitude (APA), respectively. Results are presented as mean ± standard deviation (SD) and *** represents p < 0.01 versus other groups.

Previous results from our group have shown that MSCc transplantation was able to reverse diabetes-induced prolongation of the cardiac AP at 90% of repolarization.^7^ In the present study, we observed that MSCd transplantation was also able to reverse the AP prolongation at 90% of repolarization (Figures 7A and 7B).

## Discussion

The potential of MSCc therapy in DM has been demonstrated by several groups, showing metabolic benefits such as improvements in blood glucose levels,^21,25^ renal function,^28^ neuropathy,^29^ microvascular complications,^30^ and cardiac function.^7^ However, most of these studies have used allogeneic MSC. Therefore, the use of autologous MSC from donors with diabetes should be performed to evaluate the effect on a diabetic animal model.

Our first goal was to determine how similar MSCc and MSCd were phenotypically and functionally by using *in vitro* analyses. Since both cell types could be isolated and cultivated, a full characterization was performed. These cells showed to be quite similar in several aspects, maintaining their morphological fibroblast-like shapes, mesenchymal profiles, growth kinetics, and differentiation capabilities, even at 4 weeks after diabetes induction. Aligned with our findings, previous studies have demonstrated that bone marrow MSC derived from healthy individuals compared with those derived from patients with diabetes share similar properties.^31,32^

Since changes in glucose levels could interfere with the clonogenic potential^33^ of the cells, CFU-F assays were performed. Our findings showed that cells derived from rats with diabetes had higher clonogenic potential than those derived from healthy ones. These data are in agreement with the results obtained in a previous study that demonstrated that the total number of CFU-F in diabetic male Wistar rats were higher compared with that in nondiabetic rats 4 weeks after diabetes establishment.^34^ Considering that the ability to generate clones correlates with the number of stem cells in the original sample, our data suggest that diabetic rats have a greater number of MSC than healthy rats.

The beneficial metabolic effects attributed to MSC therapy from healthy animals in a diabetic animal model have been well demonstrated by us and several other groups.^7,22,25,35^ Our findings show that MSC obtained from diabetic rats were also able to improve the metabolic profile of other diabetic rats, highlighting the therapeutic potential of these cells. As mentioned, the main causes of morbidity and mortality in patients with diabetes are cardiovascular complications.^2-4^ Thus, a previous study from our group aimed to investigate whether MSC from nondiabetic rats would reverse cardiac electrical changes induced by diabetes. The results obtained demonstrated that MSCc were able to improve diabetes-induced cardiac electrical abnormalities.^7^ In line with these results, the data presented here demonstrated that MSCd are also able to rescue cardiac AP properties impaired by the diabetic condition.

Even though the present study has a translational potential, some limitations must be considered. First, the animal model of type 1 diabetes used in this study does not exactly mimic the disease in humans. Second, the ion current contributing to ventricular repolarization in rats is different from that in humans. Third, the pathway used to deliver the vehicle in the present study (retroocular) would certainly not be appropriate for translational purposes.

## Conclusions

We characterized and showed several similarities between MSCc and MSCd derived from rats. Despite a superior clonogenic potential by MSCd, both cell types presented similar ability to restore blood glucose levels and body weight. In addition, both cells were able to reverse cardiac AP prolongation induced by diabetes. These combined *in vivo* and *in vitro* data demonstrate that MSC from diabetic animals can be an option for transplantation in diabetic animal models, indicating a potential application for humans as well.
